# Through illness, understanding

**DOI:** 10.1002/jhm.70095

**Published:** 2025-06-08

**Authors:** Matthew Bugada, Shivatej Dubbaka

**Affiliations:** ^1^ University of Cincinnati College of Medicine Cincinnati Ohio USA

## ENCOUNTER

My mind wandered as the sermon came to a close. I was halfway through my penultimate inpatient elective of medical school and was visiting my parents for Sunday breakfast and church. It was cathartic standing with them, recharging for the long week ahead. I was still tired despite my weekend off work.

“Go see if they need your help,” my dad whispered as he nudged me out of my trance. Four congregants gathered around the altar server 10 rows ahead. They half carried him to the storage room at the back of the church. I didn't know how I could help. Even if I could, I didn't have any supplies. Not to mention I wasn't even a doctor yet. Despite this, I obliged and headed toward the commotion.

The altar server sat with the others encircling him. I introduced myself as a medical student and asked if anyone was in healthcare. There was one police officer, but the rest said “no.” They explained that “Jake,” the altar server, had an unknown chronic illness.

Despite my initial hesitation, I tried to help. Pulling on my background as an EMT and medical student, I ensured his ABCs were in check, and noticed he was unresponsive to both verbal commands and even pain. The medical ID on his apple watch confirmed his chronic illness to be epilepsy, so I started timing the episode. I instructed one of the congregants to call 911 for an ambulance immediately.

Suddenly I blanked and had no idea what to do next. Thinking of my own experience with a chronic illness as a Type 1 Diabetic, I asked myself: who would know what to do next? And then it clicked, and I decided to call his mother.

In a few moments, she was able to provide critical information that allowed us to make clinical judgements with ease. First, she told me he recently changed his anti‐epileptic medications, and I silently made note of this as I considered causes of Jake's episode. Next, she stated that Jake had a rescue lorazepam, and I immediately located it in his pocket and had it readily available. As I considered whether to give Jake the rescue, I paused and asked for her advice. She said that he may not need it if he showed signs of improvement soon, but I hesitated. Would waiting prove to be the correct decision, or would my reluctance cause further consequences? I took a deep breath and thought about my own chronic illness once again. Who would I trust if I was in this situation?

Listening to Jake's mother proved to be the right course of action, as moments later Jake demonstrated symptoms of improvement on his neurological exam, sluggishly giving me a thumbs up when asked. As he regained consciousness, I talked him through the events and confided in him, “I have diabetes and was an altar server too. I would go to the backroom to check my blood sugar and treat it when it went low.” Although he couldn't respond, he offered a slight nod of understanding. In that moment, even though we didn't share the same burdens, we were brought closer by different, yet similar shared experiences.

Soon after, Jake's father arrived at the church along with the paramedics. After debriefing with the paramedics, I stepped back and sighed in relief.

As I walked away, I was tapped on the shoulder by one of the bystanders who had initially helped Jake. He told me he was also a diabetic and congratulated me on how I handled the situation. I managed a “thanks” as I wondered, *what did I even do*? I didn't give any medications, I didn't diagnose the specific episode, and all I did was talk to him and his caregiver. Despite this, it had all worked out.

## DISCUSSION

Of all my clinical rotations and experiences, this event seems to resonate with me the most. In an uncontrolled *real life* situation, I felt that I was able to use some of the medical knowledge I had piled up over the last 4 years. But, more importantly, the disease that I have been battling since fifth grade allowed me to see this patient and his family in a deeper, more human way. This experience reminded me that vulnerability and sharing our personal histories can deepen our connection with patients and help restore our sense of purpose and vitality in the profession.

Medical professionals with chronic illness provide a unique lens into patient care. Personal experiences with medical, social, and emotional aspects of chronic illness provokes empathy toward patients experiencing similar challenges. We understand, in a visceral way, what it means to live with unpredictability, dependence, and loss of control. That lived experience doesn't just make us more attentive, it also brings clarity and a renewed purpose to our work. On that day, I arrived at church mentally and physically exhausted, drained from a long stretch of clinical work. But stepping into that moment, drawing on my own history and connecting with Jake and his family, gave me an unexpected sense of clarity and purpose. I walked away renewed and more grounded in why I chose this profession. I was eager for the week ahead. My ability to connect with Jake and to act with compassion was shaped not just by medical training, but by a personal story that continues to give me strength.

However, connecting with patients through our lived experience isn't reserved just for those living with chronic illness themselves. My own family has helped me carry this diagnosis. Both my aunt, a nurse practitioner, and my brother, now a family medicine resident, have walked alongside me through the highs and lows of diabetes. Though they haven't experienced the condition firsthand, they've shared how witnessing my journey has changed their approach to patient care. It has given them a deeper understanding of what chronic illness means, not just medically, but emotionally. Lived experience can be shared in unexpected ways, and when clinicians carry these connections into the clinical space, it not only deepens their ability to listen and relate, but helps them rediscover purpose and maintain a sense of meaning in the face of medicine's daily demands.

Despite living with a chronic illness myself, I am continually moved by the vulnerability of others, and I carry their stories with me in how I care for patients. During my second year, a patient panel was hosted, and one of my classmates with ulcerative colitis was on the panel. Listening to my fellow classmate's discussion of his chronic illness left my classmates and me in awe. We didn't have any clue about the struggles he was experiencing, all while working through the rigor of medical school. In medicine, we often keep our medical conditions hidden, as there is a culture that lacks vulnerability and prizes stoicism. But when we make space for vulnerability, we create a more compassionate and inclusive profession. Embracing these diverse health experiences not only enriches how we connect with patients but also fosters a sense of shared understanding that helps us care for one another and for ourselves.

Medical education often focuses on the diagnosis and treatment of disease, but overlooks how to sit with suffering. It rarely tells us that our own experiences with illness, including their grief and fears, can be the most powerful tools we have. When we embrace these experiences, it humanizes the disease and allows us to meet patients with humility. This kind of presence not only improves care, it sustains us. In a field often marked by burnout and isolation, vulnerability can be an antidote. It reminds us that we're human first, and that our connection to others is what keeps us whole.

By sharing this story, I hope to highlight the value of embracing the lived experiences of illness in medicine as a source of strength. Too often, medical culture discourages vulnerability, yet our personal experiences shape the way we care for others in profound ways. My journey with Type 1 Diabetes has taught me that medicine extends far beyond textbooks and treatment plans; it is about understanding patients as people, acknowledging their lived experiences, and meeting them where they are. Rather than viewing my condition as a burden, I now see it as a bridge—one that allows me to connect with patients on a deeper level, advocate for their needs, and foster a more compassionate approach to care. In the end, the daily challenges of managing my diabetes are not just struggles to overcome—they are a badge of honor, a reminder of the resilience I share with my patients, and a guiding force in the kind of physician I aspire to be.

## CONFLICT OF INTEREST STATEMENT

The authors declare no conflicts of interest.

